# Mechanochemical synthesis using hypervalent iodine reagents

**DOI:** 10.3762/bjoc.22.115

**Published:** 2026-10-13

**Authors:** Nasser Amri

**Affiliations:** 1 Department of Physical Sciences, Chemistry Division, College of Science, Jazan University, P.O. Box 114, Jizan 45142, Saudi Arabia; 2 Nanotechnology Research Unit, College of Science, Jazan University, P.O. Box 114, Jizan 45142, Saudi Arabia

**Keywords:** heterocycles, hypervalent iodine, mechanochemistry, oxidative functionalization, polymerization

## Abstract

Mechanochemical activation offers a powerful means of expanding the reactivity of hypervalent iodine reagents while advancing solvent-free synthetic methodologies. This review surveys the evolution of iodine(III) and iodine(V) chemistry in the solid state, from foundational solvent-free oxidations to mechanochemical approaches that enable efficient heterocycle construction, C–H functionalization, cascade dioxygenation, fluoroiodane-mediated fluorocyclization, and redox-initiated polymerization. Mechanical energy alters aggregation, enhances reagent–substrate contact, and promotes transformation pathways not readily accessible in solution. These developments showcase the distinctive advantages of mechanochemical hypervalent iodine chemistry and its growing impact on sustainable synthetic methodology development.

## Introduction

Growing environmental awareness over recent decades has highlighted the unsustainability of many traditional chemical processes and intensified the demand for cleaner, safer, and more efficient methodologies, particularly in light of global challenges such as climate change. In this context, chemistry has come under scrutiny, driving the search for alternative modes of activation that reduce solvent use, energy consumption, and waste [1]. Alongside thermochemistry, photochemistry, and electrochemistry, mechanochemistry represents a fourth, yet historically underappreciated, mode of chemical activation, offering significant potential to align chemical synthesis with the principles of green chemistry [2–3].

Mechanochemistry, broadly defined as chemical reactivity induced by the direct absorption of mechanical energy, has roots dating back to simple grinding practices but has re-emerged in recent decades as a powerful tool for modern synthesis [4]. Enabled by technologies such as ball milling and single- or twin-screw extrusion, mechanochemical methods facilitate solvent-free or solvent-minimized reactions, often under mild conditions and without external heating, while delivering high efficiencies and unique reactivity profiles inaccessible in solution. The enhanced performance arises from improved reactant contact, increased surface area, and reduced mass-transfer limitations [5]. Although mechanochemistry is well established in fields such as crystal engineering, materials science, and formulation, its broader application in complex chemical syntheses remains in an early stage, presenting substantial opportunities to access new reaction space and advance sustainable chemical manufacturing [5–11].

Hypervalent iodine reagents have become essential tools in modern synthetic methodology due to their strong oxidative ability, versatility, diversity, low toxicity, and mild reaction profiles [12–14]. Traditionally employed in solution-phase chemistry, iodine(III) and iodine(V) reagents offer a diverse range of reactions involving oxidations, halogenations, aminations, oxidative functionalization, in addition to their unique C–C and C–heteroatom-bond-forming capabilities, including catalytic and stereoselective processes [14–16], in addition to their valuable applications as group-transfer reagents [17–19] and carbene and nitrene precursors [14,20]. In recent years, mechanochemical and solvent-free approaches have emerged as highly attractive alternatives to solution-based synthesis using hypervalent iodine reagents [21–22]. Mechanical activation through milling facilitates intimate solid–solid contact, accelerates reaction rates, and in many cases enables reactivity patterns not accessible in solution.

This review expands upon foundational solvent-free hypervalent iodine chemistry and traces its evolution through mechanochemical heterocycle synthesis, C–H functionalization, fluorination, and polymer chemistry. A very recent report on mechanochemical synthesis using hypervalent iodine reagents has emerged during the preparation of this work for submission [23].

## Review

### Combination of mechanochemistry and hypervalent iodine chemistry

Despite the tremendous growth of interest in hypervalent iodine chemistry and the resurgence of mechanochemistry over the past two decades, as reflected by the surge in publications in both fields, the integration of mechanochemistry with hypervalent iodine chemistry remains largely underexplored, even though this approach offers substantial advantages [22].

**Scheme 1:**
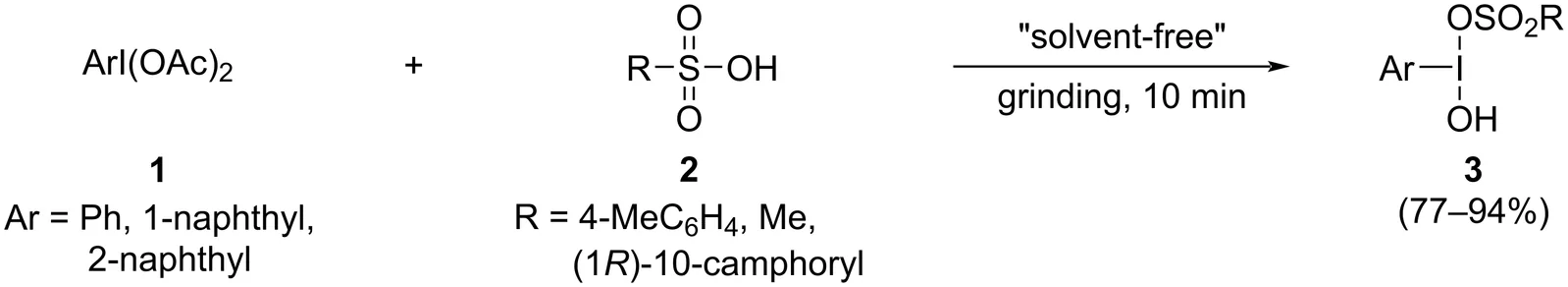
Mechanochemical synthesis of Koser’s-type reagents **3**.

Over the long history of hypervalent iodine chemistry, a wide range of preparation methods of hypervalent iodine reagents has been developed [13,24]. These methods comprise two main strategies: 1) oxidation of low-valent iodine species with chemical oxidants [25–27] or via anodic oxidation [28–30] and 2) ligand exchange of premade hypervalent iodine reagents. The vast majority of these methods are solution-based. One of the early accounts on combining the benefits of hypervalent iodine chemistry and mechanochemistry was reported by Yusubov and Wirth in the year 2005 [31]. The authors reported a solvent-free synthesis of Koser’s-type reagents **3**, simply by grinding commercially available hypervalent iodine reagents **1** such as (diacetoxyiodo)benzene (PIDA) with the corresponding sulfonic acids **2** (Scheme 1). The reactions are fast, and the yields are generally comparable or higher than those for previously reported solution-based syntheses.

α-Tosyloxylation of carbonyl compounds is a well-established model reaction in hypervalent iodine chemistry [32–33]. Using this transformation as a representative example the authors also reported one-pot mechanochemical generation, and reaction of hypervalent iodine reagents as exemplified by successful tosyloxylation of 1,3-dicarbonyl compounds [31].

**Scheme 2:**
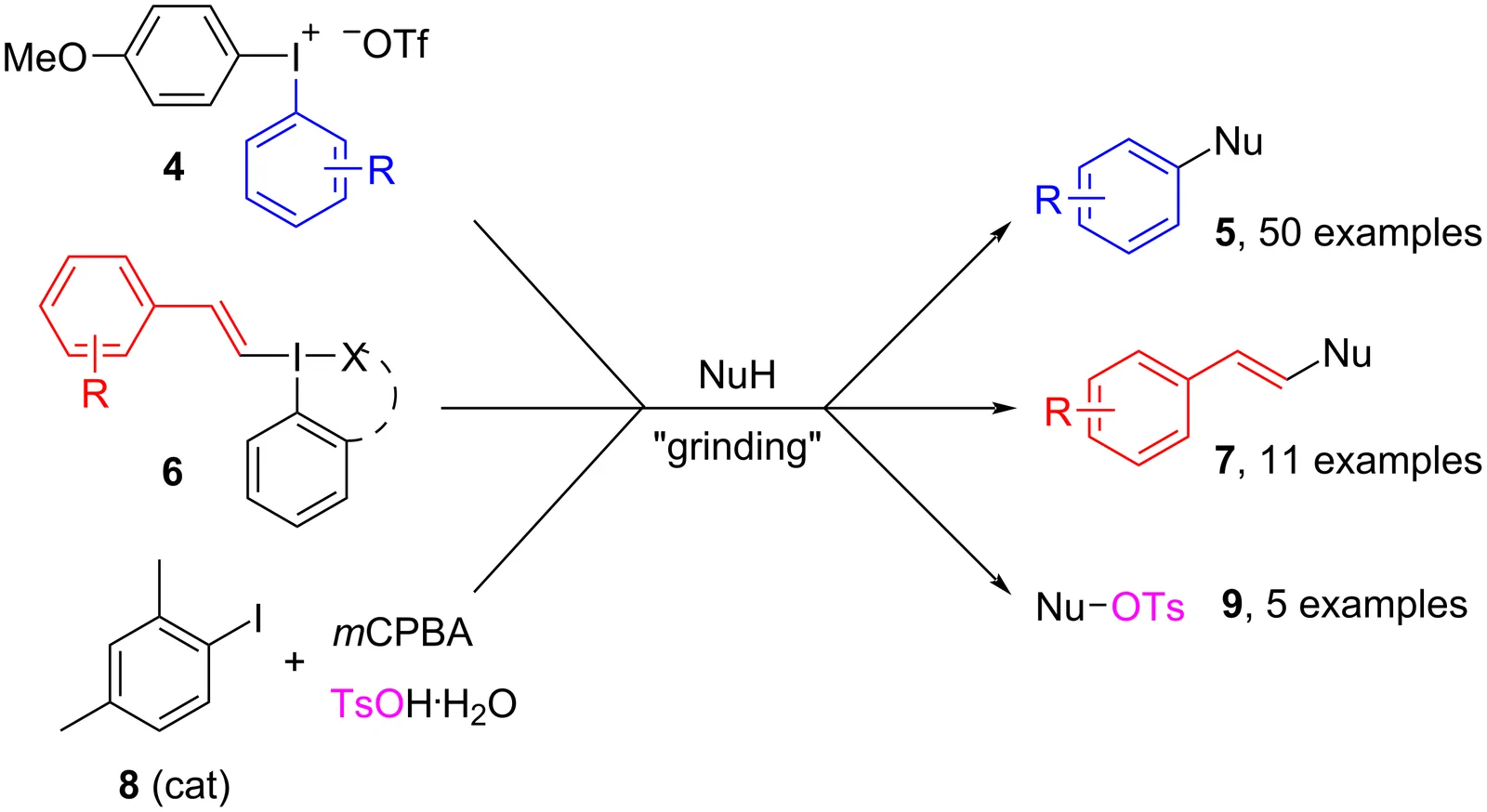
Translation of various solution-phase hypervalent iodine-mediated oxidative transformations to mechanochemistry.

Recently, Olofsson and co-workers shed more light on the topic by looking at translating various typical solution-based hypervalent iodine-mediated transformations into the realm of mechanochemistry (Scheme 2) [22]. They studied the arylation of various nucleophiles including alcohols, thiols, and nitroalkanes using diaryliodonium salts **4** and a wide range of products **5** were produced in high yields. Alkene products of type **7** were efficiently synthesized via mechanochemical vinylation of thiols and nitroalkanes using vinylbenziodoxolones (VBX, **6**). The authors also demonstrated the feasibility of catalytic hypervalent iodine-mediated oxidative transformations under mechanochemical conditions as exemplified by the successful α-tosyloxylation of carbonyl compounds (products **9**) using catalytic amounts of 2,4-dimethylphenyl iodide (**8**) and *m*-chloroperbenzoic acid (*m*CPBA) as a terminal oxidant.

**Scheme 3:**
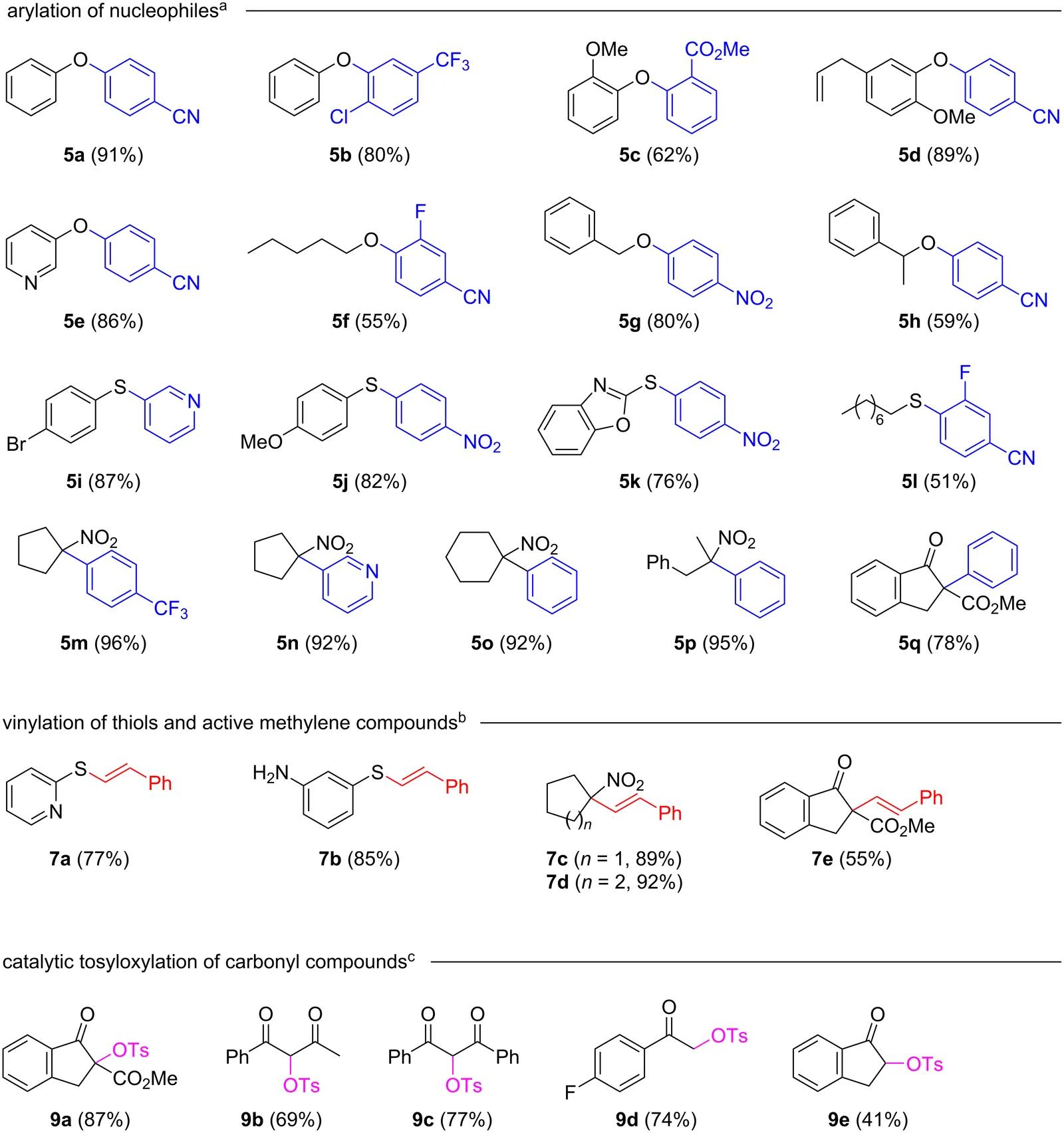
Selected examples of mechanochemical transformations using different hypervalent iodine reagents. a) **4** (0.3 mmol), alcohol (1.0 equiv), K_2_CO_3_ (1.0 equiv), LAG (EtOAc or MeCN), ball mill (35 Hz, 30–150 min). b) **6** (0.3 mmol), thiol or nitroalkane (1.0 equiv), K_2_CO_3_ (1.0 equiv), ball mill (30 Hz, 120 min). c) Carbonyl substrate (0.3 mmol), ArI **8** (20 mol %), *m*CPBA (1.0 equiv), TsOH·H_2_O (1.0 equiv), LAG (HFIP, 20 µL), ball mill (25 Hz, 5–30 min).

This study demonstrated the successful conversion of hypervalent iodine-mediated, solution-phase arylation, vinylation, and tosyloxylation reactions into efficient mechanochemical processes, supported by benchmarking and sustainability analyses. Several reagents showed notably different reactivity and regioselectivity under mechanochemical conditions. In addition, the first catalytic hypervalent iodine protocol using mechanochemistry was developed. Further, the study revealed that liquid-assisted grinding (LAG) [34–35] was beneficial in most cases. Scope studies identified multiple cases where the solid-phase methods outperformed traditional solution-based procedures, and E-factor comparisons confirmed that the mechanochemical protocols offer significantly enhanced sustainability. Some selected examples are depicted in Scheme 3 [22].

**Scheme 4:**
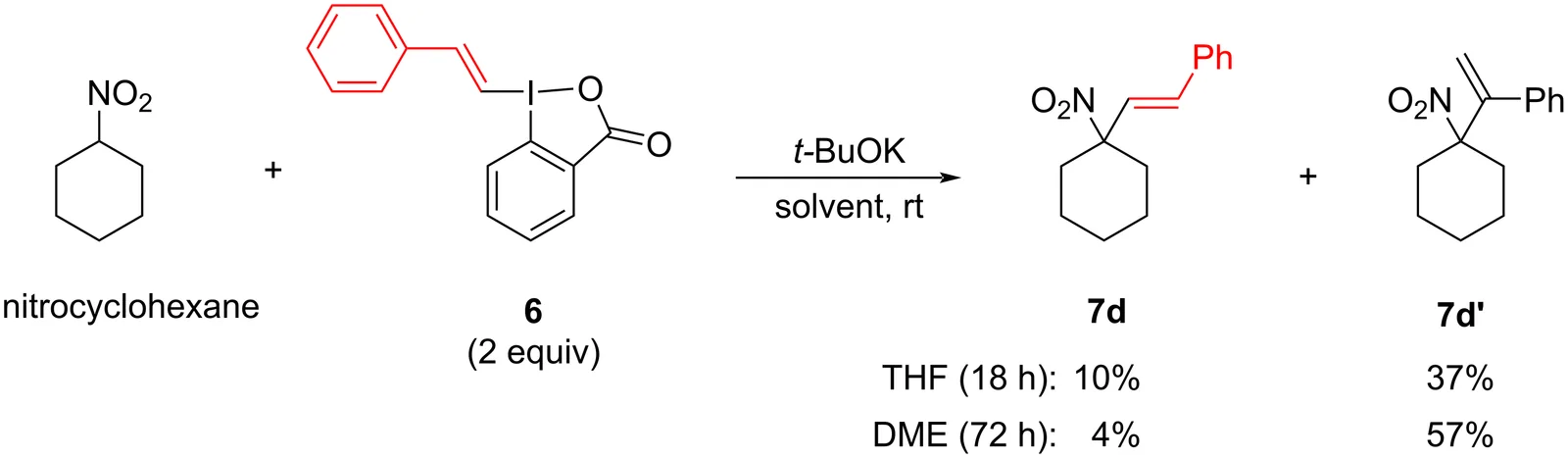
Reactivity of VBX reagent **6** towards nitrocyclohexane under solution-phase conditions.

It is noteworthy that under mechanochemical conditions, VBX reagents **6** reacted smoothly with nitrocyclopentane and nitrocyclohexane, affording the corresponding alkene derivatives **7c** and **7d**, respectively, in excellent yields and selectivity. In contrast, the same transformation under conventional solution-phase conditions required substantially longer reaction times and was sluggish giving a mixture of products with poor yields and selectivity (Scheme 4) [36–37]. This comparison clearly demonstrates the enhanced reactivity, selectivity, and reaction kinetics under milling conditions, in addition to their environmental and sustainability benefits.

### Mechanochemical synthesis of heterocycles and oxidations

Nitrogen-containing heterocycles are highly prevalent in modern pharmaceuticals, being present in approximately 82% of new U.S. FDA-approved small-molecule drugs between 2013 and 2023 [38–39], while around a quarter of small-molecule drugs contain at least one fluorine atom [40–41]. Therefore, developing synthetic methods for fluorinated heterocycles is of utter importance to the pharmaceutical industry and human health.

**Scheme 5:**
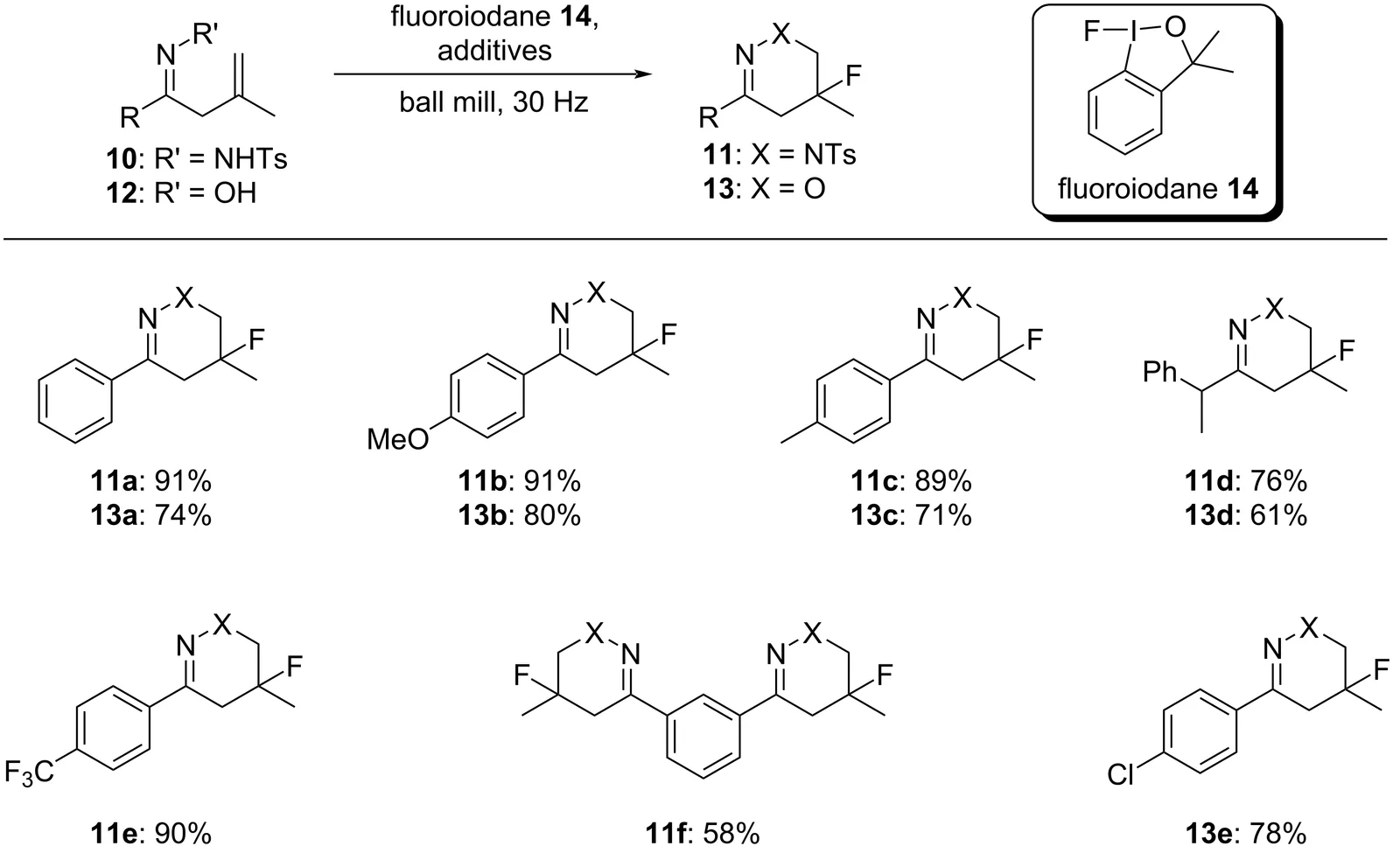
Mechanochemical intramolecular fluorocyclization of β,γ-unsaturated hydrazones **10** and oximes **12** using fluoroiodane reagent **14**. Conditions: a) Hydrazone **10** (0.25 mmol), **14** (1.5 equiv), HFIP (52 µL), ball mill (30 Hz), 15 min. b) Oxime **12** (0.25 mmol), **14** (1.5 equiv), AgBF_4_ (2.8 equiv), ball mill (30 Hz), 1 h.

Riley et al. [42] established an efficient fluorocyclization strategy for the rapid synthesis of two classes of fluorinated heterocycles: tetrahydropyridazines **11** and dihydrooxazines **13** via the reaction of β,γ-unsaturated hydrazones **10** and oximes **12**, respectively, with the readily available fluoroiodane reagent **14**. Comparable excellent yields of fluorinated tetrahydropyridazines **11** were obtained under both solution and mechanochemical conditions, while ball milling markedly enhanced the yield and selectivity for dihydrooxazine **13** formation (Scheme 5).

This work highlights the distinct reactivity relative to conventional electrophilic fluorinating agents, such as Selectfluor and demonstrates that fluoroiodane reagents such as **14** offer a complementary approach to fluorination, with mechanochemistry further revealing its capacity to efficiently enable mechanistically complex transformations.

**Scheme 6:**
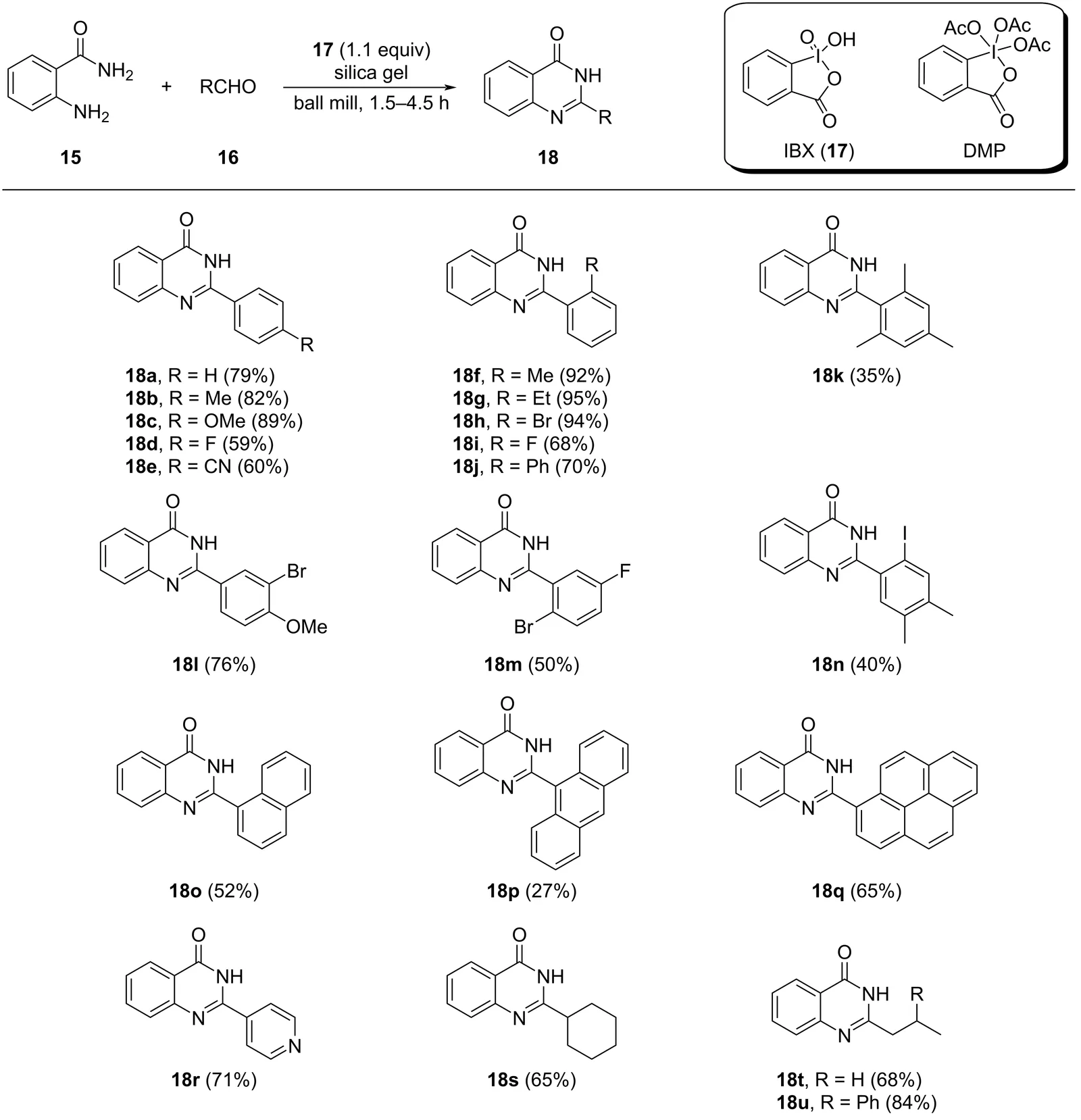
IBX-mediated mechanochemical synthesis of quinazolin-4(3*H*)-one derivatives **18**.

Direct mixing of various arylamines with hypervalent iodine(V) reagents is generally impractical due to the highly exothermic nature of the reaction, which can lead to hazardous outcomes, including explosions. During their attempt to develop a mechanochemical synthesis of quinazolin-4(3*H*)-ones, Alam et al. [43] found that grinding of a mixture of 2-iodoxybenzoic acid (IBX), aniline, and benzaldehyde is explosive. A similar result was observed when Dess–Martin periodinane (DMP) was used instead of IBX. However, introducing an *ortho*-amido substituent on anilines enables these reactions to proceed safely, as the intramolecular interaction provides effective control over reactivity. Under solvent-free mechanochemical conditions where the reactants are at maximal contact, 2-aminobenzamides **15**, aryl- or alkylaldehydes **16**, and the iodine(V) reagent *O*-iodoxybenzoic acid (IBX, **17**) undergo smooth transformation to give substituted quinazolin-4(3*H*)-ones in moderate to high yields (Scheme 6). The reaction tolerated a wide variety of aromatic, aliphatic, and heterocyclic aldehydes with various substitution patterns and varied electronic and steric effects.

**Scheme 7:**
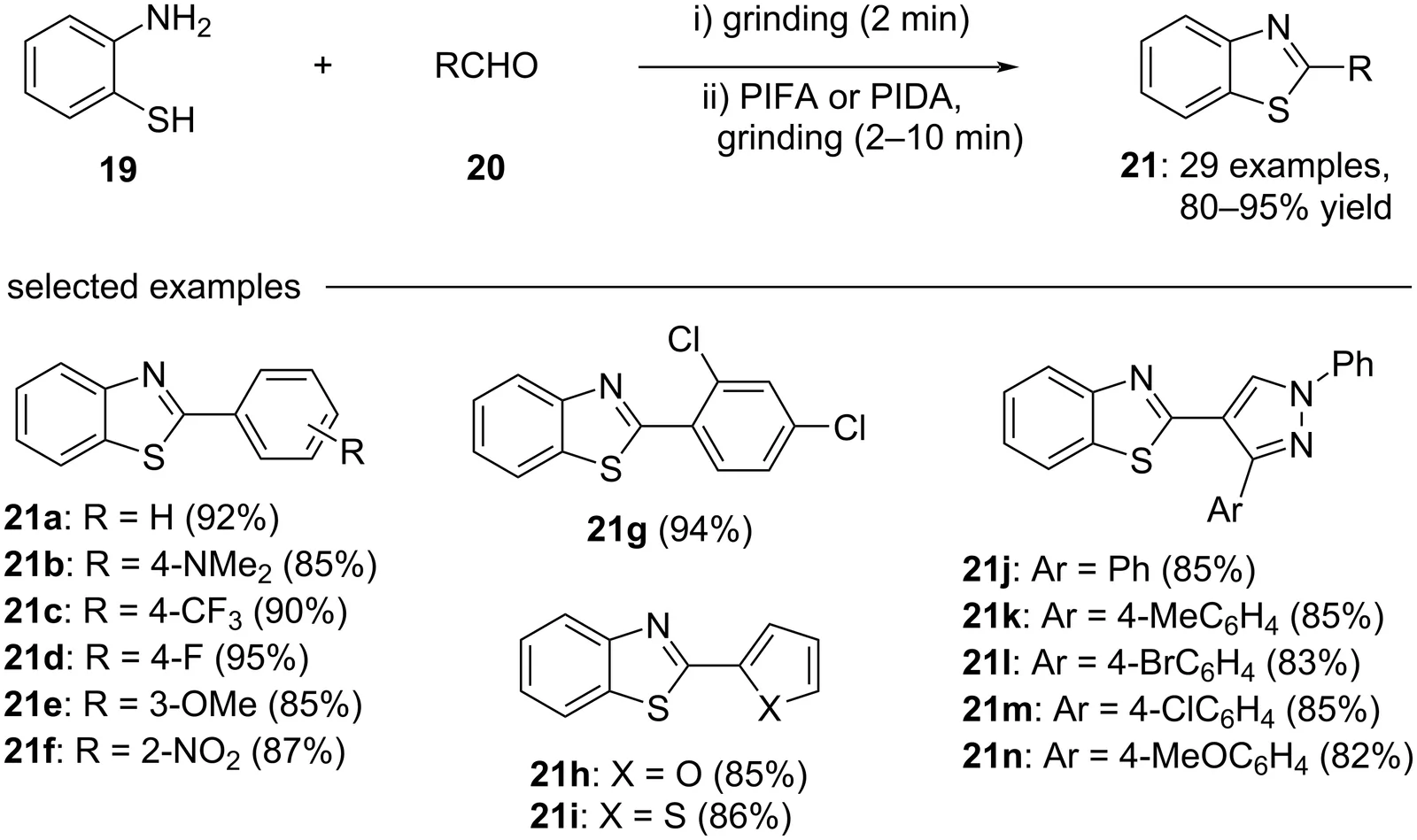
Iodine(III)-mediated solvent-free synthesis of benzothiazoles **21**.

Similarly, a straightforward, eco-friendly, one-pot approach for synthesizing 2-aryl and 2-heteroarylbenzothiazoles **21** from 2-aminothiophenol (**19**) and aryl/heteroarylaldehydes **20**, using hypervalent iodine(III) reagents under solvent-free conditions was reported by Kumar et al. [44]. The transformation is carried out simply by grinding the reactants with the iodine(III) oxidant using a mortar and pestle. Both [bis(trifluoroacetoxy)iodo]benzene (PIFA, **22**), and (diacetoxyiodo)benzene (PIDA, **23**) delivered the desired products (Scheme 7) in comparable yields with shorter reaction times in case of PIFA (**22**). The heterocyclization/oxidation process proceeds smoothly under grinding conditions, in few minutes (2–10 min).

**Scheme 8:**
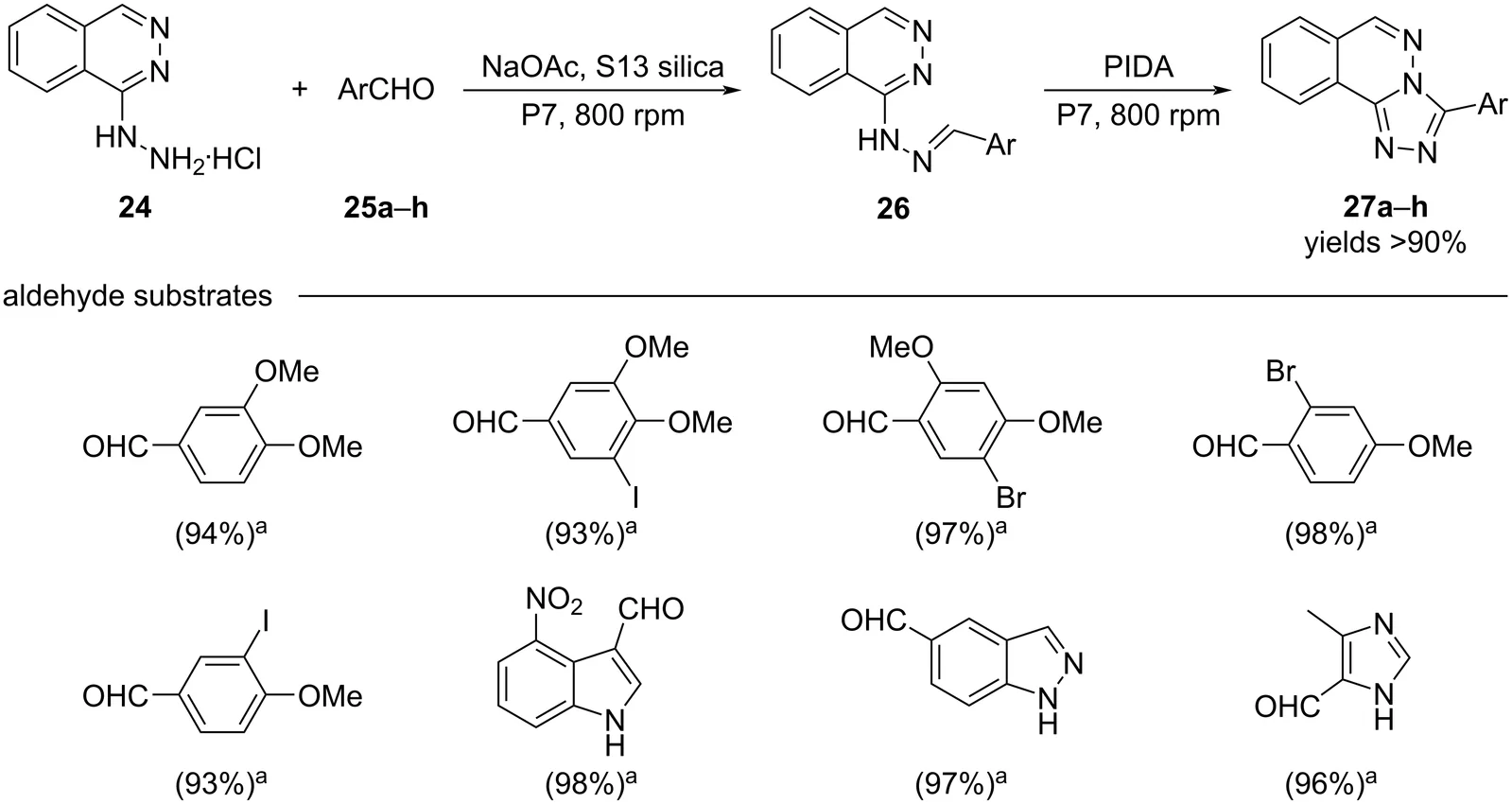
One-pot two-step mechanochemical synthesis of triazoles **27**. ^a^Overall yields of the corresponding products **27a–h**.

Furthermore, a mechanochemical synthesis of annulated 1,2,4-triazoles **27** from hydralazine hydrochloride derivatives **24** was developed using a Pulverisette 7 planetary ball mill (P7) [45]. The authors identified conditions that enable complete conversion to hydrazone intermediates **26** within minutes when pyrogenic silica (HDK^®^ S13) is employed as a grinding auxiliary, primarily facilitating efficient energy transfer. Optimal cyclization of hydrazones **26** to the triazoles **27** was achieved with (diacetoxyiodo)benzene (PIDA, **23**) for nonphenolic hydrazones (Scheme 8). Interestingly, the one-pot, two-step mechanochemical protocol was successfully demonstrated for the first time, and evaluation through green metrics confirmed its advantages over conventional solution-based syntheses.

**Scheme 9:**
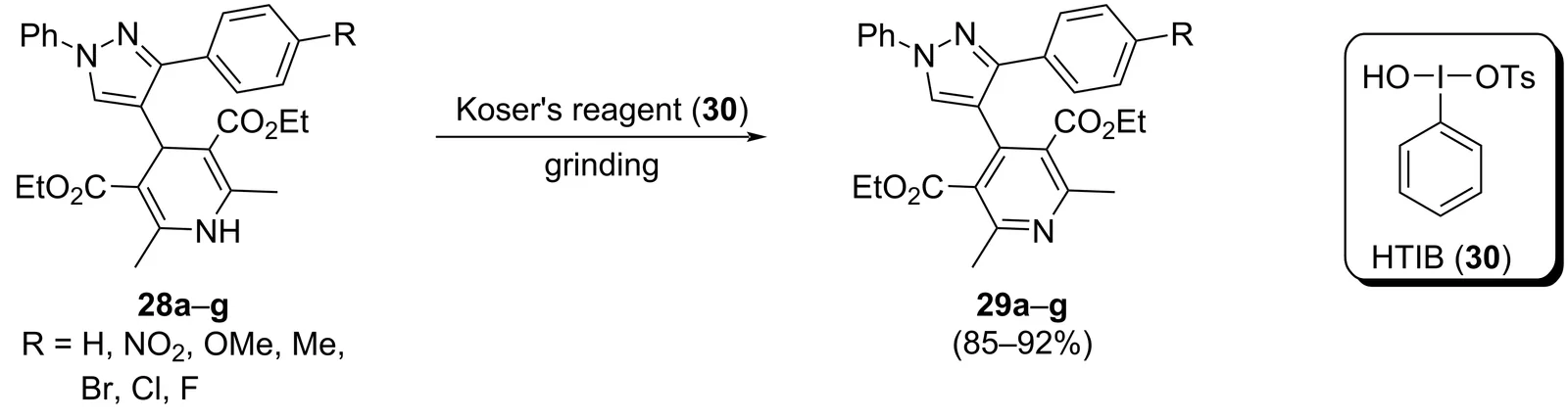
Mechanochemical oxidative aromatization of 1,4-dihydropyridines **28** using Koser’s reagent (**30**, HTIB).

In the same context, the hypervalent iodine-mediated oxidative aromatization of 1,4-dihydropyridine derivatives **28** (Scheme 9) was achieved mechanochemically furnishing the aromatized products **29** in good yields. The study [46] found that Koser’s reagent **30** – [hydroxy(tosyloxy)iodo]benzene (HTIB) – is a more efficient oxidant than PIDA and PIFA in terms of yield and time.

**Figure 1:**
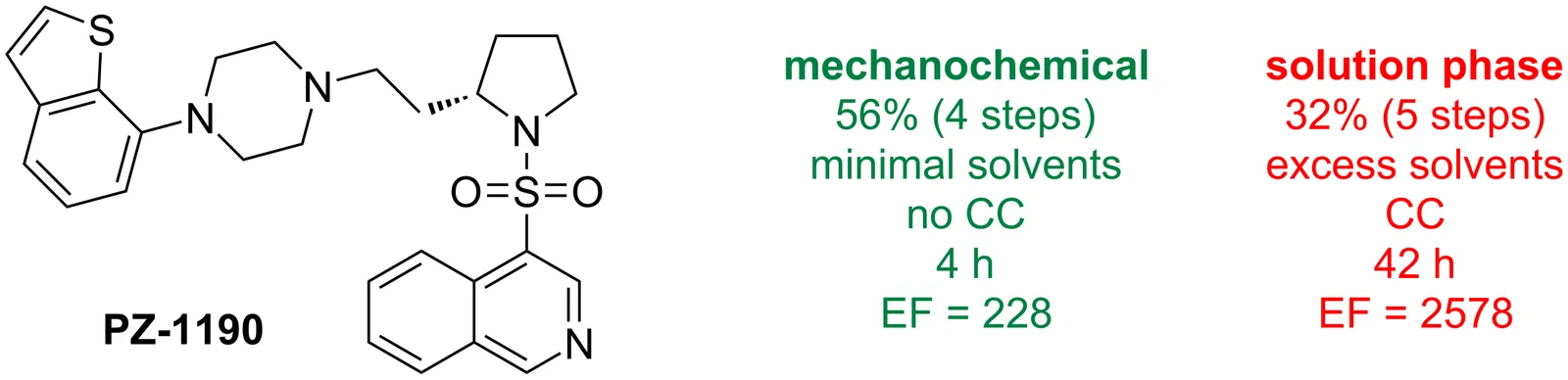
Comparison of mechanochemical and conventional solution-phase routes to **PZ-1190**. CC = column chromatography; EF = E-factor.

Canale et al. [47] reported a mechanochemical synthetic approach for the multitarget serotonin and dopamine receptor ligand **PZ-1190**, offering substantial advantages over the conventional solution-phase synthesis. The mechanochemical route reduced the synthesis from five to four steps, increased the overall yield from 32% to 56%, and shortened the reaction time from 42 h to 4 h. Reduced solvent use and elimination of column chromatography also lowered the E-factor more than tenfold, from 2578 to 228, demonstrating markedly improved efficiency and sustainability (Figure 1).

**Scheme 10:**
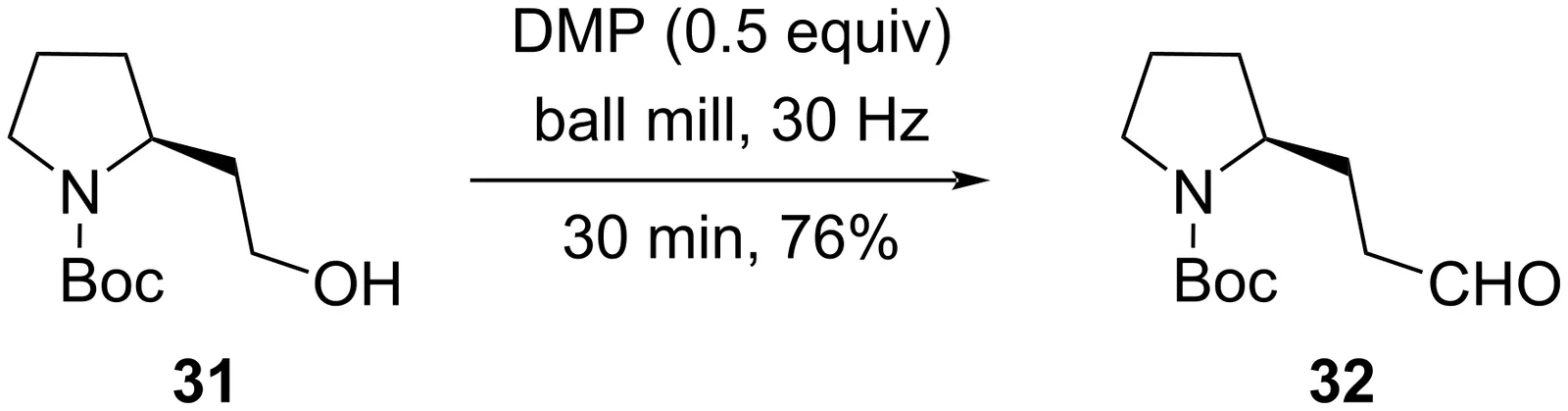
Mechanochemical oxidation of primary alcohol **31** into aldehyde **32**.

Of particular interest is the mechanochemical oxidation of the aliphatic primary alcohol **31** to aldehyde **32**, reported as the first example of this transformation under milling conditions. Milling of **31** with IBX (0.5 equiv) gave 88% conversion after 45 min, whereas DMP (0.5 equiv) afforded complete conversion within 30 min. The optimized DMP protocol was successfully performed on a 1.5 g scale, providing aldehyde **32** in 76% yield (Scheme 10).

### Mechanochemical C–H and cascade functionalization

Indoles are a fundamental heterocyclic scaffold due to their widespread presence in natural products, pharmaceuticals, agrochemicals, and advanced materials [48–50]. Although highly C2- and C3-selective transition-metal-catalyzed C–H alkynylations of indoles using ethynylbenziodoxolone (EBX) reagents are well established [47–50], their sustainability is limited by the need for large amounts of often hazardous solvents, long reaction times, and relatively high catalyst loadings.

**Scheme 11:**
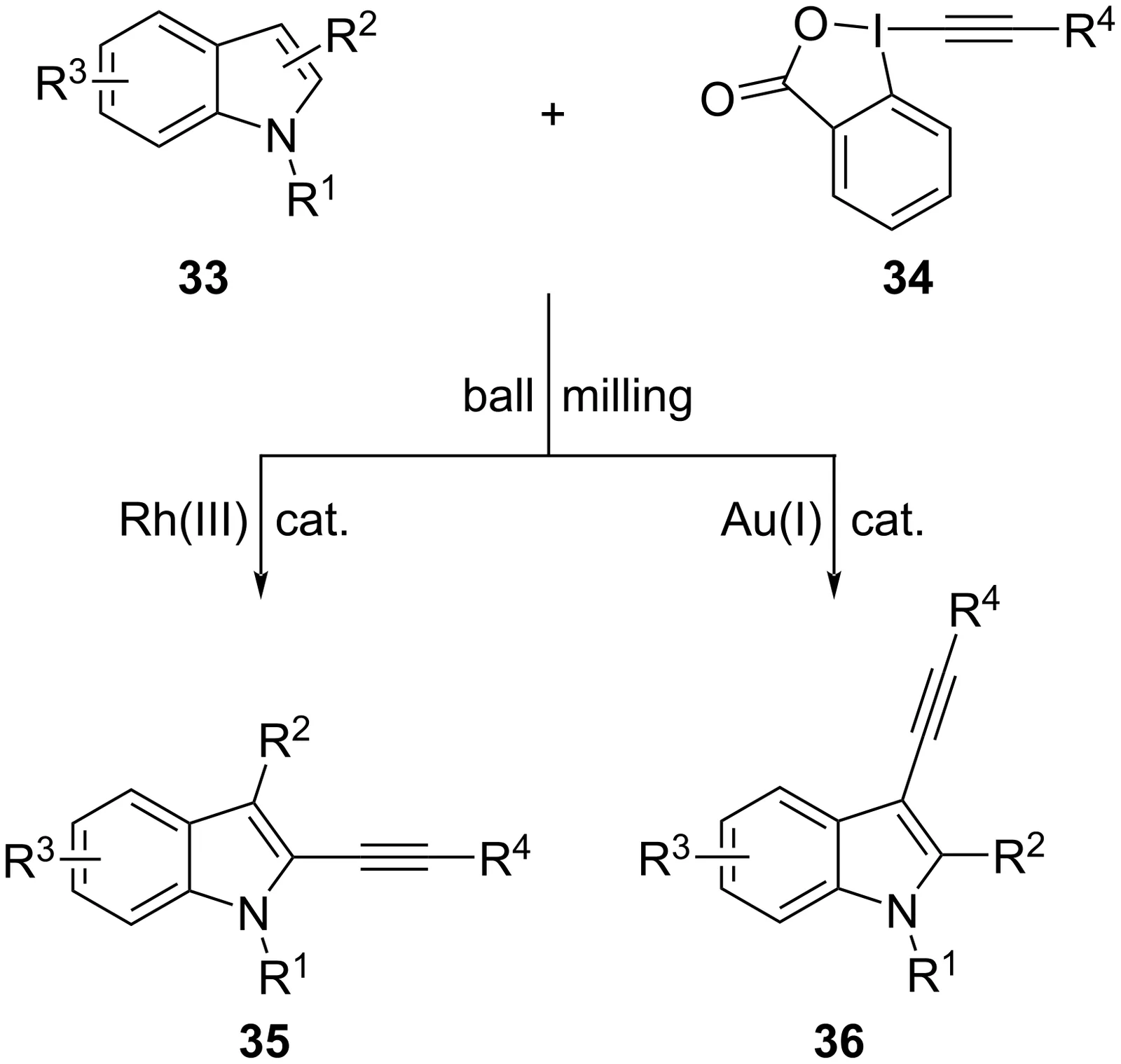
Mechanochemical C–H-bond alkynylations of indoles.

In this context, the selective mechanochemical C–H alkynylation of indoles **33** has been achieved under Rh(III) and Au(I) catalysis using ethynylbenziodoxolone (EBX) reagents **34**, enabling complementary access to C2 and C3-alkynylated products **35** and **36**, respectively, under solvent-free conditions (Scheme 11).

These mixer-mill protocols operate without external heating, tolerate a wide range of functional groups, and deliver high yields under ambient atmosphere. Relative to conventional solution-phase methods, the mechanochemical approach significantly reduces reaction times and catalyst loadings, leading to enhanced turnover frequencies, highlighting its efficiency for metal-catalyzed C–H functionalization reactions [51].

**Scheme 12:**
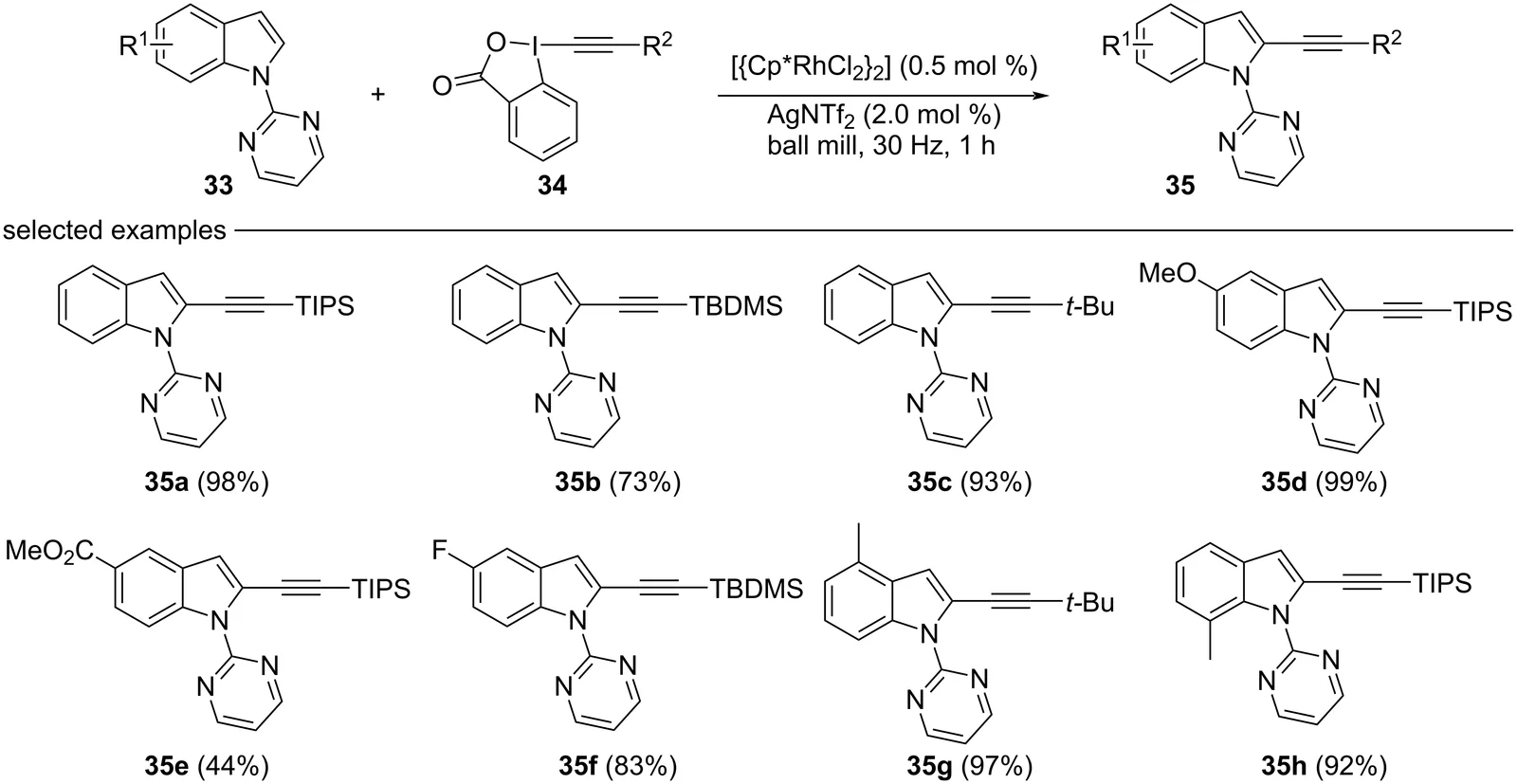
Selected scope of mechanochemical Rh(III)-catalyzed C–H alkynylation. TIPS = triisopropylsilyl, TBDMS = *tert*-butyldimethylsilyl.

Under Rh(III) catalysis, the reaction is C2-selective leading to C2-alkynylated indole products **35** in generally excellent yields using low catalyst loading (0.5 mol %). Some selected examples are presented in Scheme 12.

**Scheme 13:**
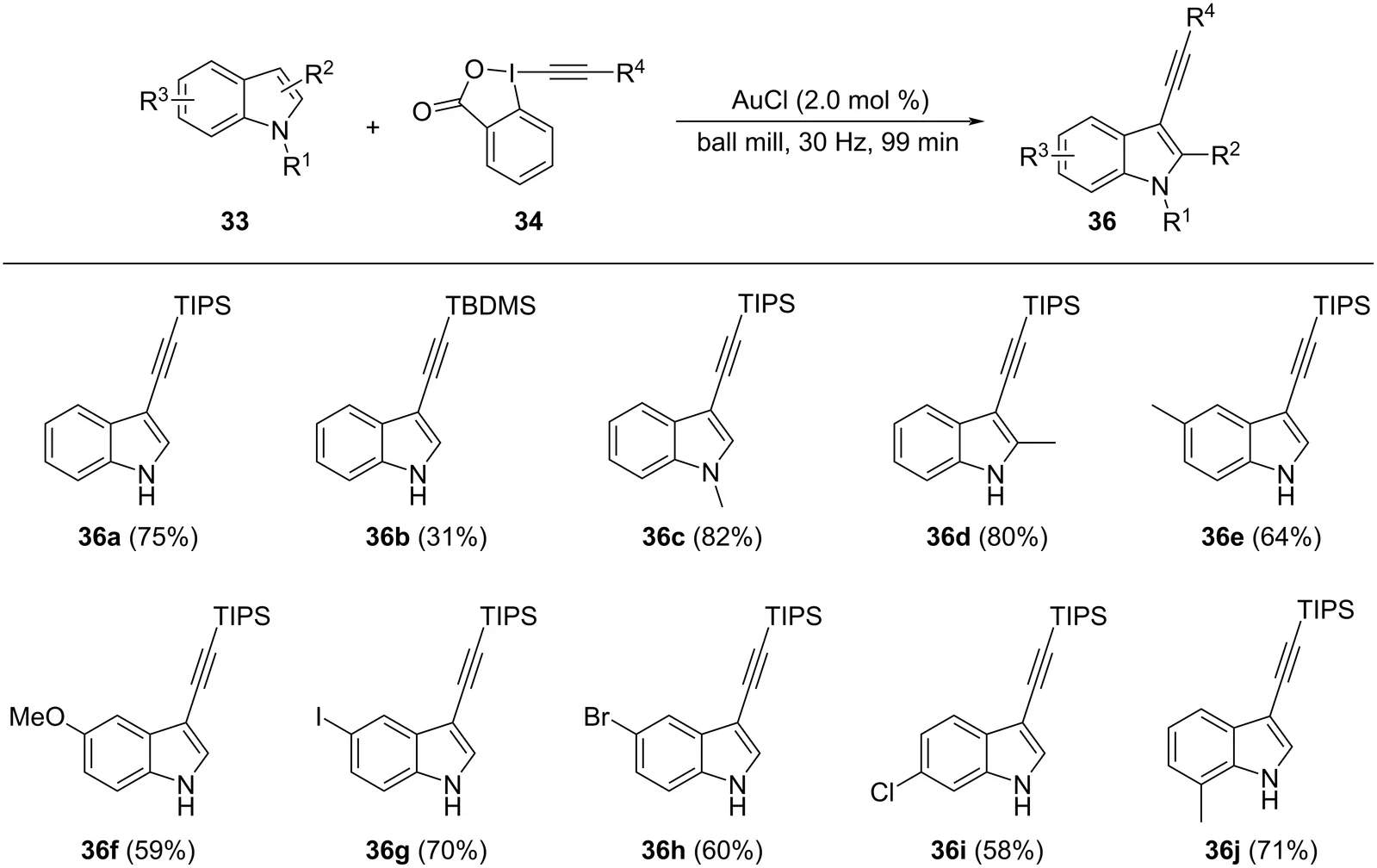
Scope of mechanochemical Au(I)-catalyzed C–H alkynylation. TIPS = triisopropylsilyl, TBDMS = *tert*-butyldimethylsilyl.

While under Au(I) catalysis, the reaction is C3-selective, producing C3-alkynylated indole products **36** in generally good yields (Scheme 13).

**Scheme 14:**
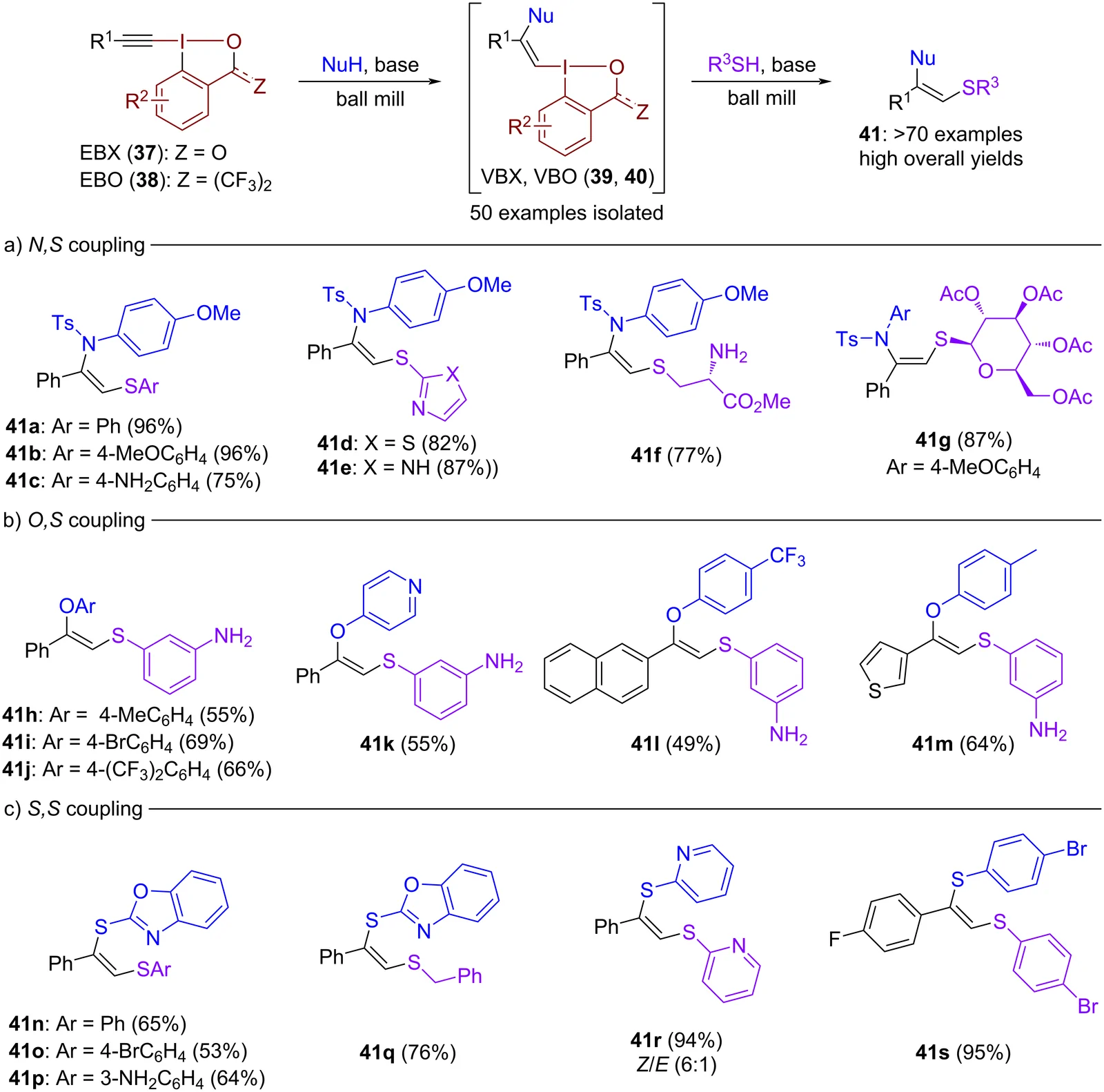
Mechanochemical synthesis of complex alkenes.

Highly substituted alkenes are versatile intermediates for molecular diversification, and recent advances have shown that nucleophilic addition to vinylbenziodoxol(on)es (VBX/VBO) enables their synthesis with excellent regio- and stereocontrol. Building on this, Olofsson and co-workers [21] recently reported a transition-metal-free mechanochemical strategy that directly converts ethynylbenziodoxol(on)es (EBX/EBO) **37** and **38** into 1,2-heteroatom-substituted (*Z*)-alkenes **41** in a one-pot process under solvent-free conditions, bypassing the isolation of VBX/VBO **39** and **40** intermediates. This approach delivers a broad range of *N*-, *O*-, and *S*-VBX reagents in high yields and, through a telescoped nucleophilic addition, provides access to numerous novel and complex (*Z*)-alkenes with excellent functional group tolerance, including bioactive motifs such as glucose and cysteine, some of which are depicted in Scheme 14. Benchmarking against solution-phase methods demonstrates that this tandem transformation is inefficient or inaccessible in solution, underscoring the unique capability of mechanochemistry to enable otherwise impractical synthetic pathways.

**Scheme 15:**
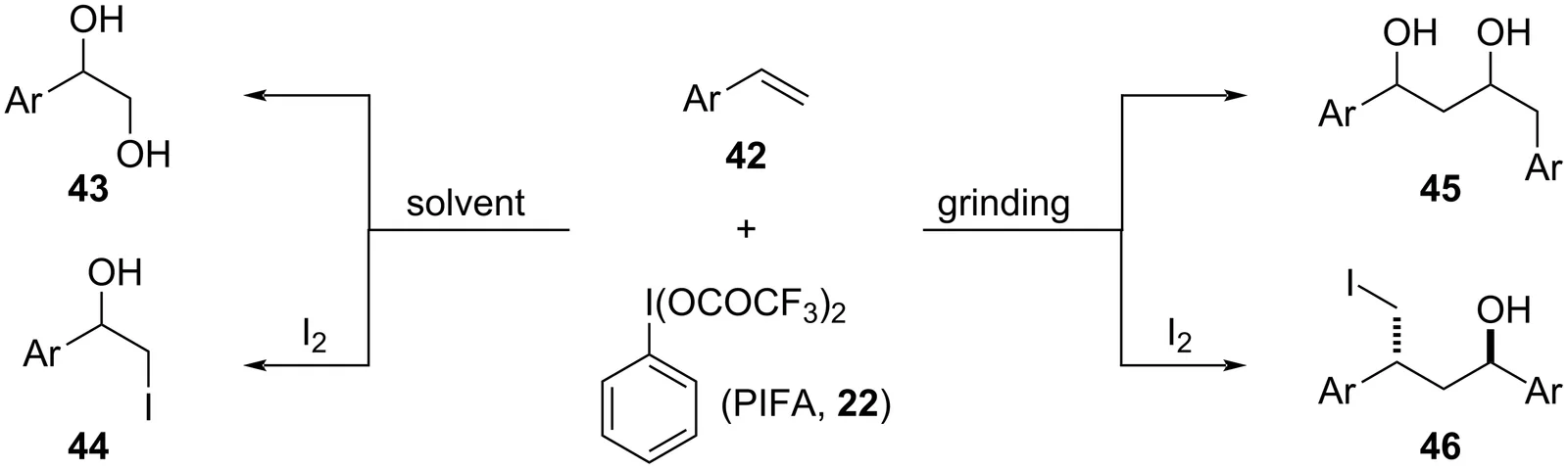
Mechanochemical PIFA-mediated cascade functionalization of styrene derivatives.

Furthermore, a mechanochemical strategy for the cascade functionalization/dimerization of styrene derivatives **42** using PIFA (**22**) has been established [52], providing efficient access to 1,3-dioxygenated products **45**. Under closely related conditions, the inclusion of molecular iodine enables the formation of 1,4-iodoalcohols **46**. These synthetically valuable motifs are difficult to obtain using conventional solution-phase methodologies, that usually deliver the 1,2-difunctionalization products **43** and **44** (Scheme 15).

**Figure 2:**
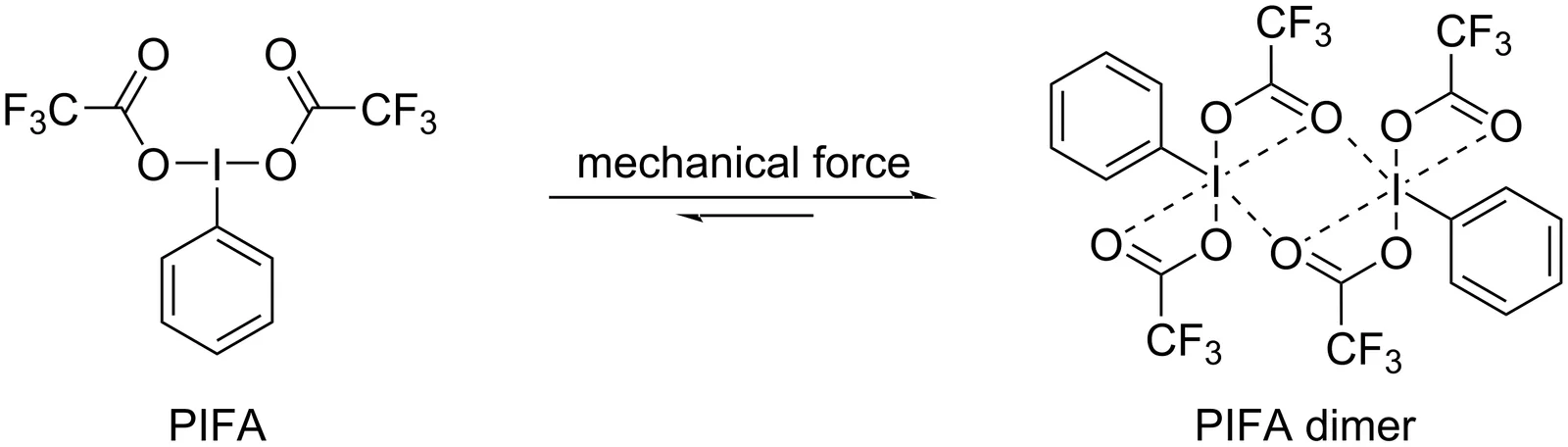
Mechanochemical dimerization of PIFA.

To understand the different reaction outcomes under mechanochemical and solution-phase conditions, the authors performed several control experiments and mechanistic studies. They attributed the divergent selectivity to the possible preferential formation of PIFA dimer under mechanical force (Figure 2). This association is proposed to alter the availability of reactive PIFA species and favour a cascade pathway that ultimately delivers the 1,3-disubstituted product. By contrast, in solution, the greater availability of free trifluoroacetate is proposed to direct the reaction through a substitution pathway, accounting for the conventional 1,2-dioxygenation selectivity.

### Mechanochemical *N*-functionalization reactions

Diaryliodonium salts are air- and moisture-stable solids that have been extensively studied since the late 19th century, with rapid growth in their synthesis and applications over the past decade. Their low toxicity and high reactivity make them excellent electrophilic arylation reagents, enabling cross-coupling with arenes, alkynes, alkenes, carbonyl compounds, and heteroatoms [53–58]. However, traditional methods often require long reaction times, and sometimes complex ligands which limits their broader use. Therefore, developing more efficient methods is of great interest.

**Scheme 16:**
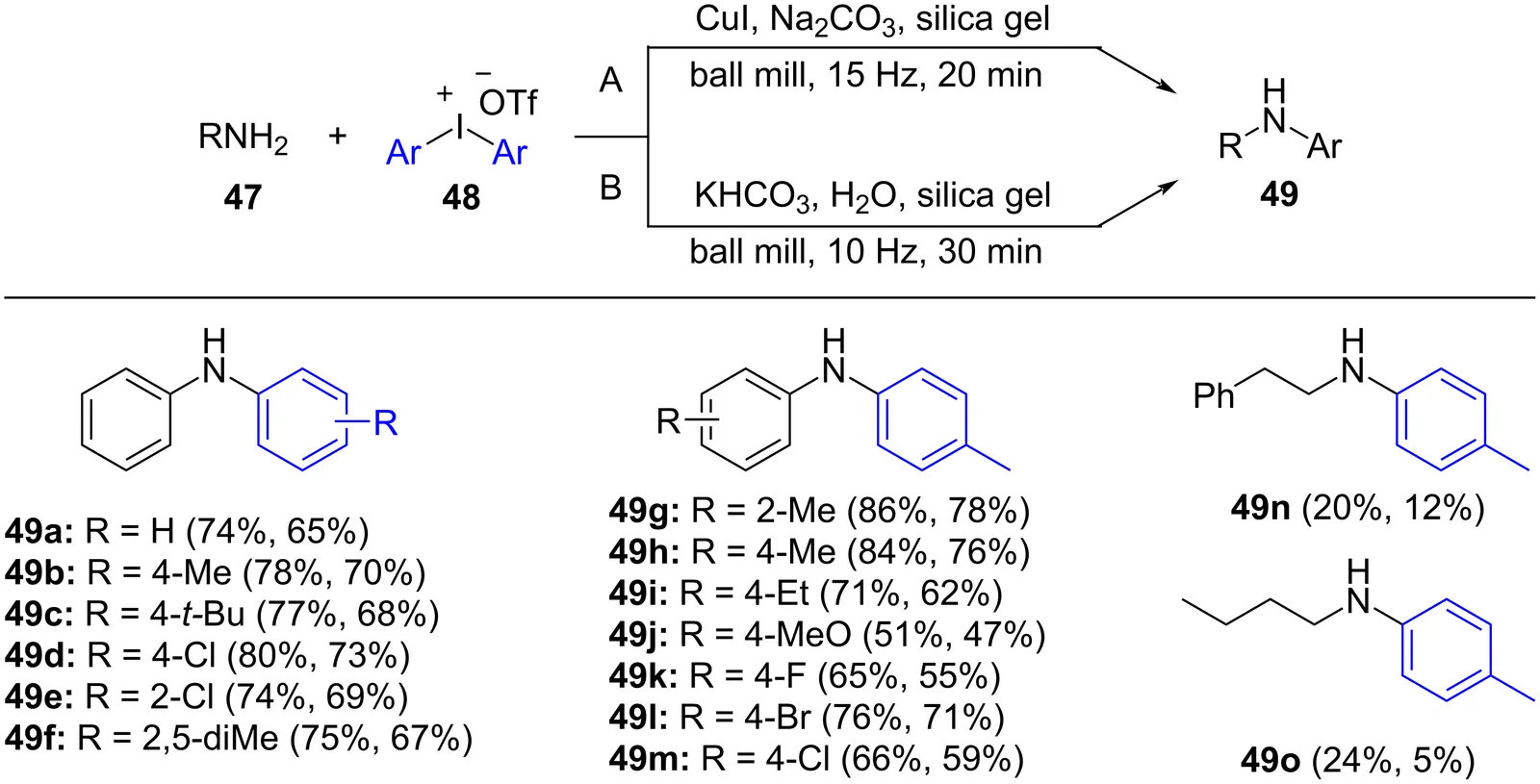
Mechanochemical arylation of amines with diaryliodonium salts. Yields correspond to method A and B, respectively.

**Figure 3:**
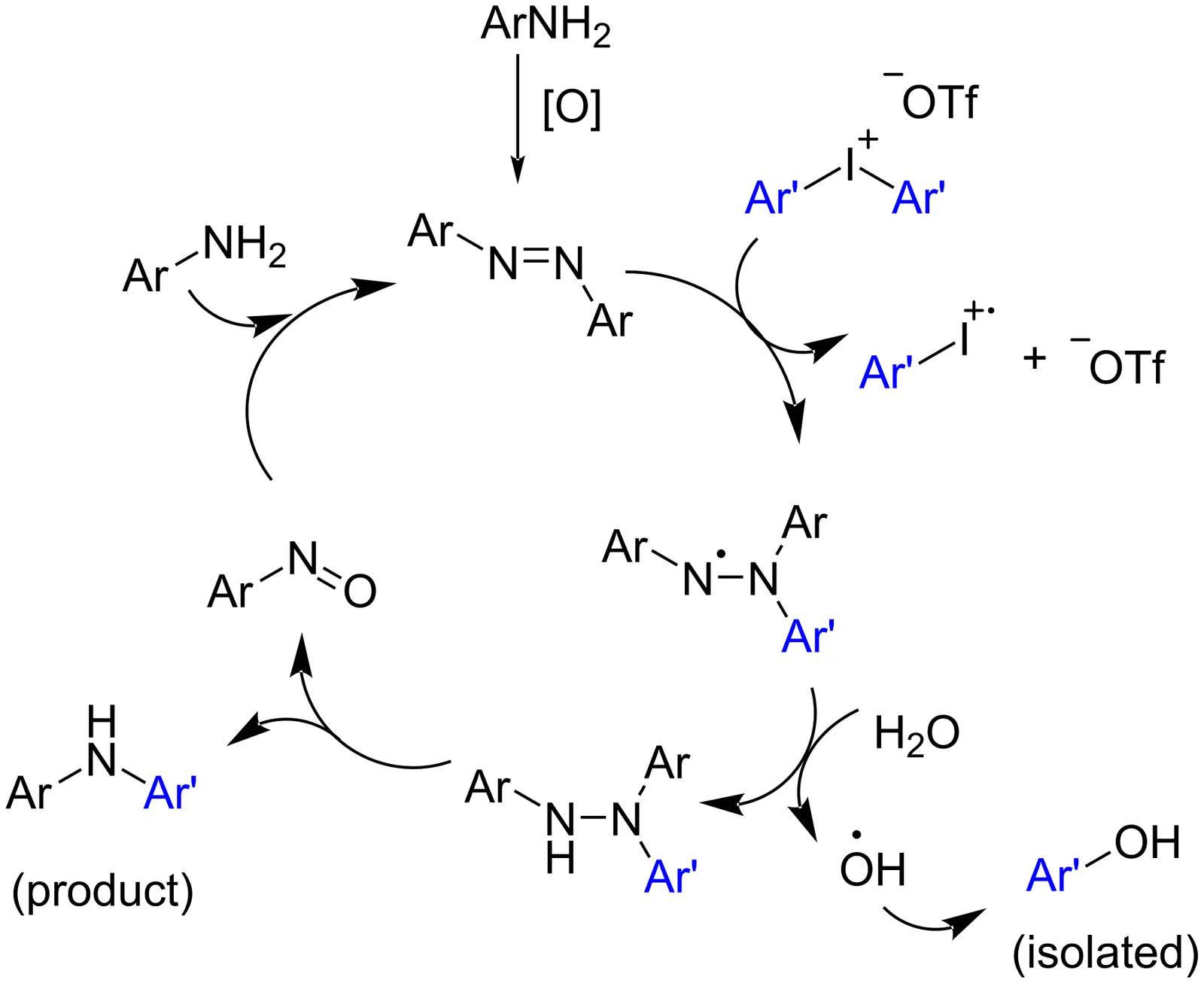
Proposed radical mechanism for the formation of diarylamines (method B).

As mechanochemical methods are known to improve reaction kinetics relative to their solution counterparts, Jiang and Li [59] studied the mechanochemical *N*-arylation of amino compounds **47** using diaryliodonium salts **48** (Scheme 16). They demonstrated that diaryliodonium salts are highly effective for *N*-arylation under mechanochemical conditions. When combined with Cu(I) catalysts (method A), the reaction proceeded smoothly without solvent, achieving high yields in case of aromatic amines but suffered from diminished yields in the case of aliphatic amines. The authors also developed a Cu-free version of the reaction. In the absence of the Cu catalyst, liquid-assisted grinding (LAG) was necessary to achieve yields comparable to the Cu-catalyzed protocol, therefore the reaction benefitted from the addition of small amounts of water (method B). Mechanistic investigations revealed that the reaction proceeds via a radical mechanism in the absence of the metal catalyst (method B) as depicted in Figure 3.

**Scheme 17:**
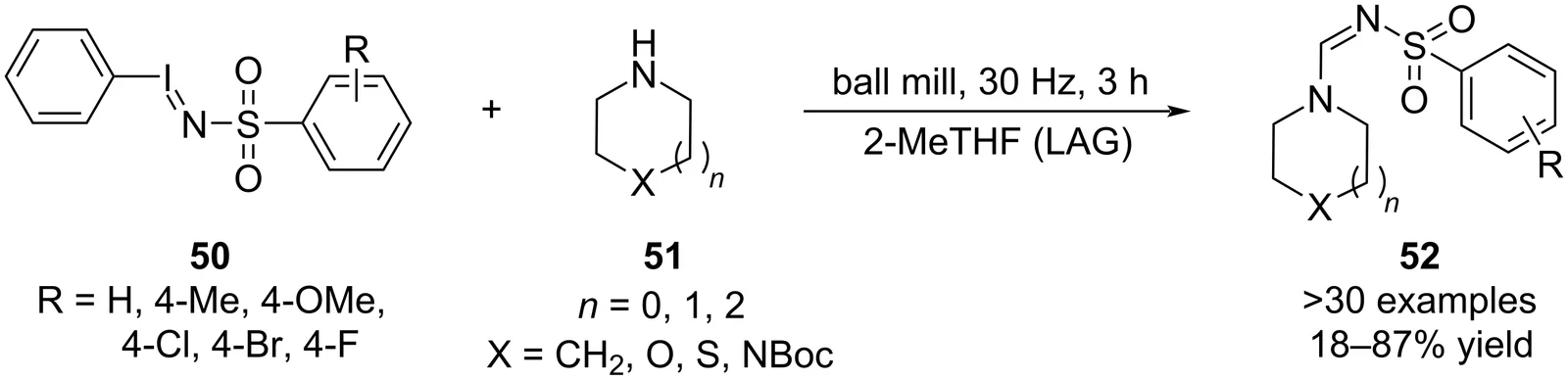
Mechanochemical synthesis of *N*-sulfonylamidines.

Guha et al. [60] developed an operationally simple, metal-free mechanochemical protocol for the *N*-sulfonyl transfer from various iminoiodinanes **50** to a range of cyclic secondary amines **51** affording *N*-sulfonylamidines **52** in yields ranging from low to high (Scheme 17). The reactions were carried out in a ball mill with minimal amounts of 2-methyltetrahydrofuran (2-MeTHF) as LAG additive. The transformations proceeded efficiently at ambient temperature, typically reaching completion within 3 hours, and required no metal catalysts or bases. As *N*-sulfonylamidines are valuable building blocks in natural products and drug intermediates [61–62], this method provides a practical and scalable (gram-scale demonstrated) approach to their synthesis.

### Mechanochemical polymerization

The ability of iodonium salts to generate radicals under thermal, photochemical, or redox conditions found various applications in organic synthesis. Diaryliodonium salts are extensively studied as photoinitiators in photopolymerization: a process that is essential for 3D-printing and various other applications.

**Scheme 18:**
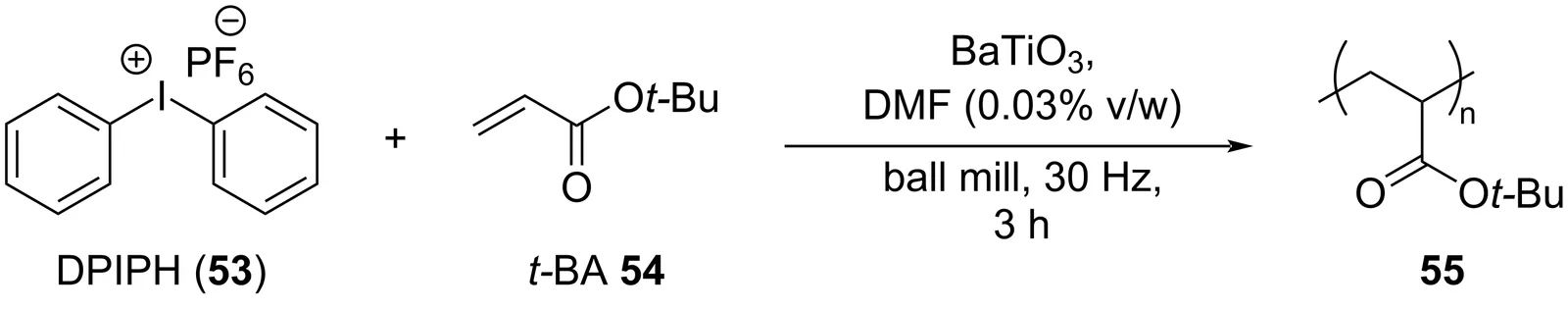
DPIHP as initiator for the FRP of acrylate monomers.

In 2022, Zeitler et al. [63] explored the potential of mechanochemical activation of diaryliodonium salts as initiators for free radical polymerization (FRP). The authors investigated the activation of diaryliodonium salts by both solution-based ultrasonication and liquid-assisted grinding (LAG) [34–35] in a ball mill. The study revealed that grinding a mixture of *tert*-butyl acrylate (*t-*BA, **54**), diphenyliodonium hexafluorophosphate (DPIHP, **53**), BaTiO_3_, and a minimal amount of DMF (0.030 v/w) leads to the formation of the desired polymer **55** with high efficiency: >95% conversion of the monomer (*t-*BA) after 3 hours only (Scheme 18) compared to 20 hours needed to achieve the same level of conversion in solution under ultrasonication. The polymerization of other monomers such as methyl acrylate (MA), ethyl acrylate (EA), butyl acrylate (BA), and methyl methacrylate (MMA) was successful in all cases under the developed mechanochemical conditions.

**Scheme 19:**
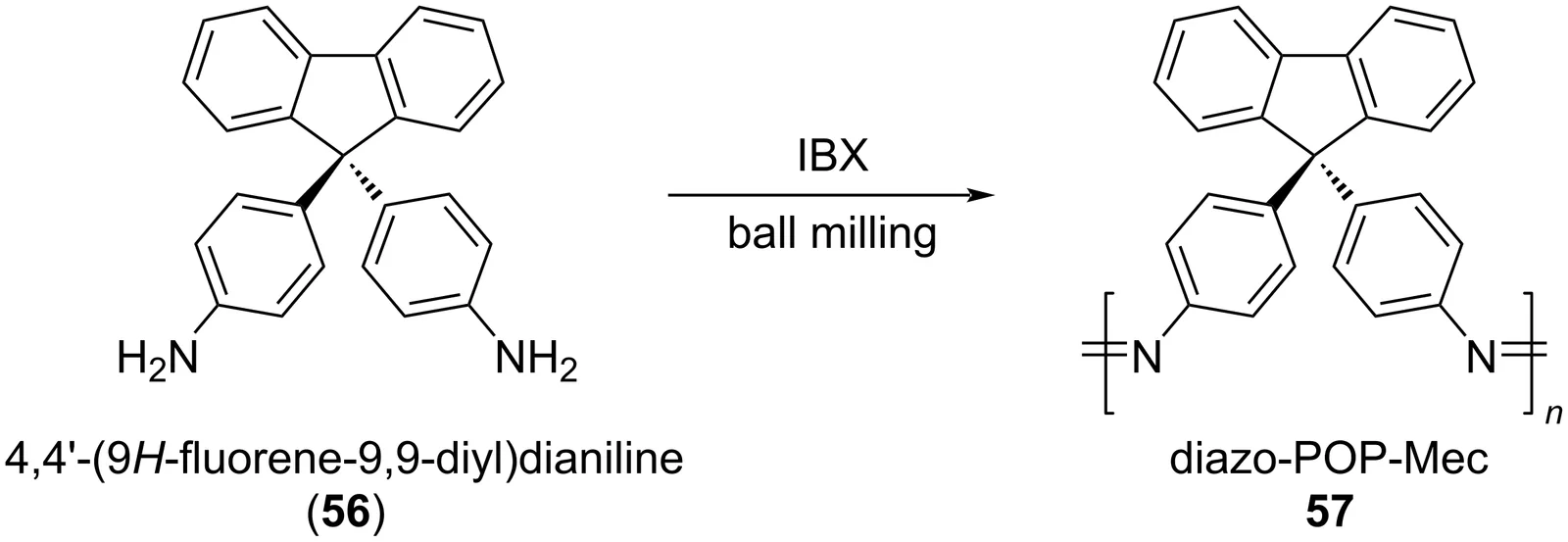
Mechanochemical synthesis of Diazo-POP-Mec **57**.

Azo-linked porous organic polymers (POPs) are an important class of materials, yet their preparation traditionally depends on metal-catalyzed, high-temperature, liquid-phase methods. Recently, Zhang et al. [64] developed a mechanochemical, oxidative and metal-free strategy for their solid-phase synthesis, using increased steric hindrance in the monomers to prevent explosive interactions between the hypervalent iodine oxidants and primary amines. The oxidative coupling of 4,4’-(9*H*-fluorene-9,9-diyl)dianiline (**56**) via ball milling proceeds smoothly using 2-iodoxybenzoic acid (IBX), without explosion or hazardous exothermic interactions to produce the desired polymer **57** (Scheme 19).

This process produced the target polymer diazo-POP-Mec **57** in just 20 minutes, which is highly efficient compared to 72 hours under solution conditions. This approach also offers soluble azo-based POPs that can be cast into an organic membrane. Overall, the results highlight the stabilizing effect of steric hindrance during azopolymer formation and demonstrate mechanochemistry as a safe and effective alternative to conventional synthesis routes.

## Conclusion

Across all classes of reactivity – oxidation, cyclization, C–H activation, fluorination, and polymerization – mechanochemistry has proven to be a powerful platform for hypervalent iodine chemistry. These solid-state transformations are not merely solvent-free analogues of solution reactions; instead, they often display enhanced reaction kinetics, selectivity, modified mechanistic pathways, and the ability to access otherwise inaccessible transformations. The body of work summarized here demonstrates how mechanical force enables new reactivity modes, simplifies operations, improves sustainability metrics, and expands the reach of iodine(III) and iodine(V) reagents into new domains such as materials chemistry. Continued development in mechanistic understanding, reactor design, and scalable processing will further unlock the potential of hypervalent iodine chemistry under mechanochemical conditions.

## Data Availability

Data sharing is not applicable, as no new data were generated or developed in this study.
